# A Machine Learning Model to Predict Knee Osteoarthritis Cartilage Volume Changes over Time Using Baseline Bone Curvature

**DOI:** 10.3390/biomedicines10061247

**Published:** 2022-05-26

**Authors:** Hossein Bonakdari, Jean-Pierre Pelletier, François Abram, Johanne Martel-Pelletier

**Affiliations:** 1 Osteoarthritis Research Unit, University of Montreal Hospital Research Centre (CRCHUM), Montreal, QC H2X 0A9, Canada; hbonakda@uottawa.ca (H.B.); dr@jppelletier.ca (J.-P.P.); 2 Medical Imaging Consultant, ArthroLab Inc., Montreal, QC H2X 0A9, Canada; francoisabram@aim.com

**Keywords:** cartilage volume loss, bone curvature, osteoarthritis, prediction, machine learning

## Abstract

The hallmark of osteoarthritis (OA), the most prevalent musculoskeletal disease, is the loss of cartilage. By using machine learning (ML), we aimed to assess if baseline knee bone curvature (BC) could predict cartilage volume loss (CVL) at one year, and to develop a gender-based model. BC and cartilage volume were assessed on 1246 participants using magnetic resonance imaging. Variables included age, body mass index, and baseline values of eight BC regions. The outcome consisted of CVL at one year in 12 regions. Five ML methods were evaluated. Validation demonstrated very good accuracy for both genders (R ≥ 0.78), except the medial tibial plateau for the woman. In conclusion, we demonstrated, for the first time, that knee CVL at one year could be predicted using five baseline BC region values. This would benefit patients at risk of structural progressive knee OA.

## 1. Introduction

Osteoarthritis (OA) is the most prevalent musculoskeletal disease and a common joint degenerative disease. OA is a global health burden and is accountable for substantial health costs [1,2]. It is characterized by chronic pain and functional disability, and the knee is the most affected among the joints [3]. The hallmark of the disease is the loss of a joint tissue, the cartilage [3].

OA diagnosis often occurs late, i.e., when the destruction of articular tissues has reached a late stage. This is of importance as although OA is characterized by being a disease of “older age”, younger people are more and more being affected by this disease [4]. Moreover, its two most prominent risk factors, age and body mass index (BMI) [5], are also of considerable concern for the healthcare system, as there is a growing number of aging and obese people worldwide who will soon confront the system with an unsustainable draw for OA individuals. Above all, there is not yet a curative cure (in the form of disease-modifying OA drugs [DMOADs]) for this disease [3,6]. Currently, OA treatments only relieve symptoms.

To be able to combat the rise of this disease, there is a critical need to identify, at an early stage, individuals at risk of having a structurally progressive disease, i.e., rapid degradation of cartilage. Indeed, therapeutic strategies used early during the pathological process may permit to reduce/stop the structural progression of the disease. In turn, this would lead to an improvement of the symptoms. This is important, as in recent years there has been an issue about the safety of some of the symptom relief treatments, which were related to potential detrimental systemic impacts such as cardiovascular risks, increased risk of morbidity, and even mortality [7,8]. Moreover, the identification of individuals at risk of having a structural progressive disease is also of high significance for the development of DMOADs. Hence, a great part of the challenge in the development of such drugs is often the inclusion of patients in trials with advanced OA (severe cartilage loss), making it difficult to reduce or stop the degenerative process, therefore not suitable for DMOAD therapy, and impeding the power analysis of such trials.

Early identification of OA structural progressors currently depends on clinical judgment with the help of radiographic evaluation. However, it is well known that X-rays are not sensitive enough to detect early knee articular alteration [9,10]. Therefore, it is of great importance to develop automated and practical tools that will identify, at an early stage, OA patients for whom articular tissue alterations will progress rapidly.

A variety of fluid biomarkers has been evaluated for such discrimination. However, despite a significant body of research in this field, there is not yet a validated signature for early diagnosis or prognosis of the disease [11]. Limitations with fluid biomarkers include, among others, the fact that there is often no direct correlation with joint structural changes, the poorly defined association with age-related changes, some being related to obesity and cannot distinguish between OA and obesity, and that the use of only one fluid biomarker cannot fully reflect the complex patterns underlying this disease.

At present, for optimal forecasting of joint structural alterations, increasing evidence points toward the use of articular structural (tissue) markers. At first, cartilage alteration was evaluated as a marker for the knee. However, when cartilage begins to show degradation as evaluated by clinical features and/or radiography it is already at a moderate stage of the disease. Recently, the change in knee bone was suggested as an accurate marker to identify early OA structural progressors; knee bone alteration was shown to precede cartilage losses and contribute to the development of the disease [12,13,14,15,16,17].

Over the years, many methodologies were introduced to evaluate such bony changes and included bone attrition, joint incongruity, periarticular area, shape, and curvature [13,14,16,18,19,20,21,22,23,24]. However, some used radiographic determination, which could lead to imprecision due to its dependence on the acquisition method and/or statistical modelling involving a component that is operator-dependent, which may introduce errors. Others used magnetic resonance imaging (MRI), and among the developed technologies, certain had shortcomings. For example, for the bone area, the assessment is subjective with inconsistent associations with knee structural progression. Machine learning (ML) techniques, coupled with MRI, have opened new possibilities for large-scale data integration to assess precise measurements of OA status in a multidimensional manner. Recently, by using these two methodologies (MRI and bone change), the measurement of the bone shape vector [25] and the subchondral bone length (SBL) [26] were reported. Yet, the bone shape vector was developed only for one bone, the femur, and included in its measurement the osteophytes (bony projections), which may induce inaccuracy in bone shape measurement changes, while the SBL uses 2D shape measurement. Another MRI fully automated methodology was developed and assessed the bone curvature (BC) [20]. This BC assessment methodology in addition to being quantitative, is patient-based, and, while preserving the measured bone surface, did remove two bone alterations (peripheral osteophytes and bone marrow lesions [BML], including edema and cysts) that could interfere with the bone measurement [20,27]. By using this system, BC alteration was shown to precede cartilage volume loss (CVL), in addition to predicting the effectiveness of OA treatment [20].

In the search for a model/tool that could offer an objective and quantitative assessment in the early forecasting of knee OA structural progressors, we hypothesized that knee BC features at baseline could predict, for an individual, CVL at one year. A primary concern was the understanding of which bone regions can play an effective role in such a prediction. Second, was the developed model able to predict CVL at one year in more than one knee subregion with the same baseline variables. Third, could the developed model be accurate for both genders, and fourth, could it be replicated and extended to another OA cohort for the prediction of outcomes? To answer these questions, we (i) applied feature selection by using ML algorithms on a fairly large sample to find the most important BC regions, (ii) developed advanced gender-based prediction models that provide high prediction performance on all the cartilage regions, and (iii) evaluated the reproducibility of the developed models by using an external cohort of OA patients from a clinical trial. Data revealed that the combination of five knee BC region values at baseline could predict CVL after one year on 12 knee regions with high accuracy and reproducibility. 

## 2. Methods

### 2.1. Study Population

The models were developed using individuals from the Osteoarthritis Initiative (OAI) cohort. The OAI cohort, an observational study of the natural progression of knee OA, included men and women between the ages of 45 and 79, enrolled at four centers across the United States (Columbus, OH; Baltimore, MD; Pawtucket, RI; Pittsburgh, PA). The cohort included 4796 individuals at baseline (https://nda.nih.gov/oai/study-details, last accessed date: 25 October 2019). For this study, 3395 participants, at the baseline, having the parameters for the classification of participants into structural progressors or no-progressors (see below for description), were included.

To validate the developed models, an external dataset consisting of knee OA patients from a clinical trial was used [28]. This cohort comprised patients with primary symptomatic knee OA from a multicenter, randomized, double-blind clinical trial evaluating the effect of Licofelone (a lipoxygenase/cyclooxygenase inhibitor). Here, 77 patients were selected from the comparator arm (Naproxen, a cyclooxygenase inhibitor) of this trial. This cohort was named Naproxen.

### 2.2. Classification of Participants into Structural Progressors

This study was performed using the structural progressors, as we wanted to develop a model on individuals presumed as having disease progression. To this end, each participant was assigned a label for their probability values of being structural progressors (PVBSP), as previously described [29]. In brief, the PVBSP label for each participant included the values of five features at the baseline, as well as an outcome. The features were two X-rays: the medial minimum joint space width (JSW) and medial joint space narrowing (JSN) as a score [30], and three quantitative MRIs: mean cartilage thickness of peripheral, medial, and central tibial plateaus. The outcome was JSN ≥ 1 at 48 months. For discrimination of the structural progressor from the no-progressor, a binary classification in the context of multilabel classification was calculated by employing a threshold value using the maximizing F1 score, as described [31].

Data revealed that for the OAI cohort, 39% of the participants were classified as structural progressors (1246; 659 women and 587 men) and used to build the model. For the Naproxen cohort (validation), these proportions were reversed, and 69% (53; 20 women and 33 men) of the patients were labelled structural progressors.

### 2.3. Knee MRI Tissue Acquisitions

For the OAI cohort, MRIs were acquired with a 3T apparatus (Magneton Trio, Siemens, Germany) using a double-echo-steady state (DESS) imaging protocol, as per the OAI protocol. For the Naproxen cohort, the MRI acquisition was done as previously described, with a 1.5T apparatus with an integrated knee coil using 3D fast imaging with steady-state precession (FISP) with water excitation (Siemens, Erlangen, Germany) or spoiled gradient echo recalled (SPGR) with fat suppression (General Electric, Milwaukee, WI, USA) [28].

### 2.4. Bone Curvature

Bone curvature was evaluated using a fully MRI automated quantitative system, as previously described [20]. In brief, the mean curvature of a surface corresponds to the average of the two eigenvalues of the Weingarten matrix and is expressed as m^−1^. The method used the cylindrical coordinate representation of the surfaces obtained by automatic segmentation [27], smoothed using a Gaussian filter of standard deviation sigma = 4 and size 6 * sigma in the configuration space, allowing for a curvature map of average resolution of 2 mm in the image by 6 mm transversely to the images. For each knee bone surface, the mean curvature was computed and averaged for all the samples of a region. In this study, the knee BC regions used as variables (input) included eight “basic” regions: lateral and medial trochlea, lateral and medial central condyle, lateral and medial posterior condyle, and lateral and medial tibial plateau. These regions were named basics, as added together, they provided the global knee or subregions.

### 2.5. Cartilage Volume and Loss

The cartilage volume was measured using, as previously described and validated, an automated (OAI) [32,33] or a semi-automated (Naproxen) [34,35,36] human knee cartilage segmentation. The percentage CVL was calculated as follows: (cartilage volume at one year—baseline cartilage volume/baseline cartilage volume) × 100. Twelve global or regional CVL at one year were evaluated and included: (i) global knee, femur (trochlea + condyle), condyle, and tibial plateau; (ii) lateral compartment (femur + tibial plateau), femur (trochlea + condyle), condyle, and tibial plateau; (iii) medial compartment (femur + tibial plateau), femur (trochlea + condyle), condyle, and tibial plateau. In this study, CVL at one year was chosen, as it was the smallest elapsed time that could reliably measure the change using MRI methodologies.

### 2.6. Model Development

**Figure 1:**
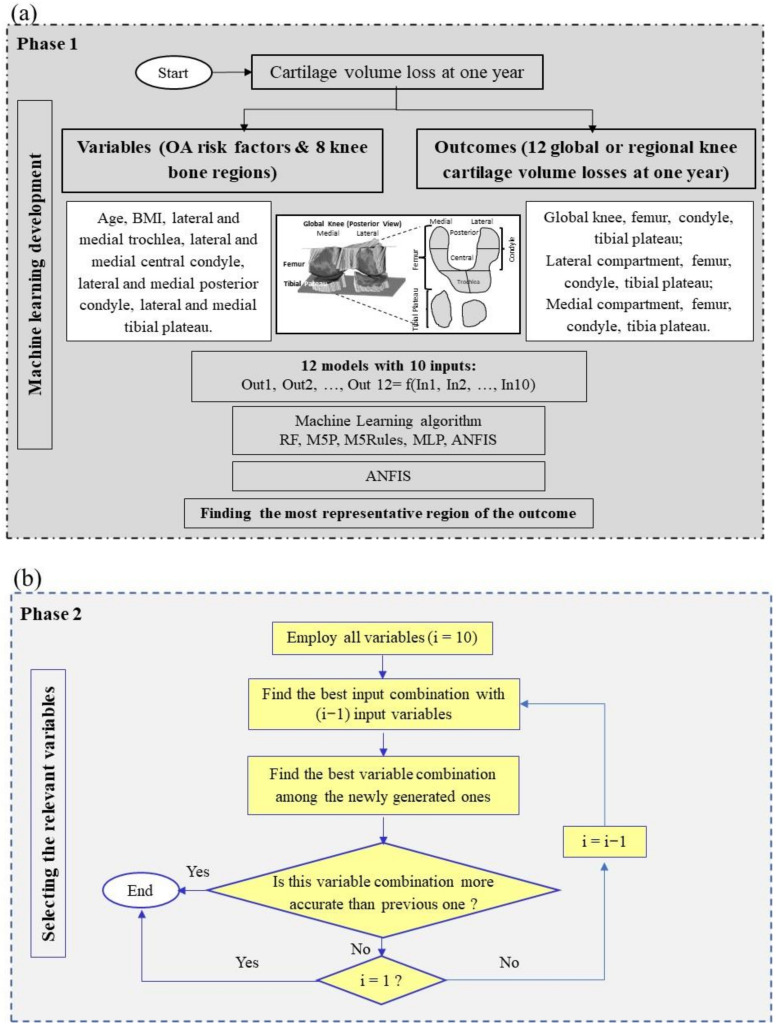
Applied methodology in machine learning development. The development of the prediction model was performed in two phases (**a**) Phase 1 and (**b**) Phase 2 as described. Outcome (Out)1, Out2, …, Out12 represent 12 global or regional knee cartilage volume losses at one year; In1, In2, …, In10 represent two OA risk factors and eight knee bone curvature regions. BMI, body mass index; RF, random forest; M5P, M5 prime; MLP, multilayer perceptron; ANFIS, adaptive neuro-fuzzy inference system.

The development of the prediction model was performed in two phases (Figure 1). As illustrated in Figure 1a, Phase 1, the independent variables (input) based on gender separation were grouped into two major OA risk factors (age and BMI) and eight knee BC regions, and the outcomes (output) were the CVL at one year in 12 regions. After selecting the best ML algorithm, the most representative region of CVL at one year as the outcome was identified. Further, and as illustrated in Figure 1b, Phase 2, the relevant input variable combination was identified.

#### 2.6.1. Phase 1

##### Selecting the Best ML Algorithm

Five different ML-based methods were investigated. The ML techniques included tree- or non-tree-based methods. The tree-based methods were random forest (RF) [37], M5Rules [38], and M5P [38], and the non-tree-based methods were multilayer perceptron (MLP) [39] and the adaptive neuro-fuzzy inference system (ANFIS) [40]). The outcomes of ML analysis with tree- and non-tree-based methods and statistical analysis were implemented using MATLAB and Waikato Environment for Knowledge Analysis (WEKA) software. The main concept of each method is provided in the Supplementary Materials Table S1.

##### Finding the Most Representative Region of CVL at One Year as the Outcome

In contrast to the custom ML problem using one outcome as the target, this study was confronted with 12 outcomes, which was a challenging task. Our strategy was to find, as a first step, the most representative region to develop a model, and then assess the algorithm of the developed model with the other 11 regions. To this end, the best ML algorithm was used to analyze each outcome region (CVL at one year) using three different statistical indices (see below Statistical analysis).

#### 2.6.2. Phase 2

##### Selecting the Variable Combination

With the use of the representative cartilage region as the outcome, we further investigated the most influential variable combinations. Selecting the relevant variables in ML models saves resources in the data collection step during model development or model applications. Having fewer misleading variables not only improves the accuracy of the ML model but removes multicollinearity that reduces the possibility of overfitting in the ML model. The variable reduction was performed stepwise, in which each step included the reduction of one and then two variables. This strategy not only removed the lowest-cost variable(s) among all possible input combinations but also led us to check the synergy of two variables along with variable reduction. In total, with ten input variables, 1023 different input combinations can be defined; however, by applying the above-mentioned strategy, 97 different variable combinations were evaluated.

##### Systematic Controllability Variable Reduction

We then performed the systematic controllability variable reduction for CVL prediction at one year. We had ten variables, then the process of variable reduction started with nine and eight variables for which all possible combinations were analyzed. The best model from this step was employed in the next step of variable reduction. This process of reducing variables continued until the ML results showed a significant decrease in accuracy, and the reduction in the number of variables did not further reduce the ML modelling accuracy.

### 2.7. Statistical Analysis

To evaluate the performance of the different methods with different variable combinations, three statistical indices were employed. They included correlation coefficient (R) as a correlation-based index, root mean square error (RMSE) and mean absolute error (MAE) as two well-known absolute indices. The simultaneous use of these indices to verify the efficiency of a model provided a robust evaluation [41].

## 3. Results

### 3.1. Participant Characteristics

A comparison between the structural progressor baseline characteristics of OAI with Naproxen (Table 1) showed that OAI participants had lower BMI, WOMAC scores, JSW, and BC in the medial compartment. Moreover, OAI participants had higher cartilage volume in the global and lateral compartments. These indicate that the patients from the Naproxen cohort had a higher level of disease severity, which also explained the higher amount of structural progressor participants in the Naproxen (69%) compared to the OAI (39%) cohorts.

### 3.2. Gender-Based Model Development

#### 3.2.1. Finding the Optimal Parameters for Each of the ML Methods

Five well-known ML techniques in solving complex nonlinear problems were evaluated: M5P, RF, M5Rules, MLP, and ANFIS. The optimal values of the parameters for each ML technique were found through a trial-and-error process and are described in the Supplementary Materials, Table S1.

#### 3.2.2. Selection of ML Technologies

Next, the best ML-based modelling algorithm was investigated. All ten variables (risk factors and BC regions) were employed to estimate 12 outcomes (CVL regions) using the five mentioned ML-based models. Results showed (Supplementary Materials, Table S2) for all the outcomes that ANFIS had higher or equal accuracy (R) and lower or equal RSME and MAE than the other ML methodologies, except in one region (lateral tibial plateau for the MLP, where the difference for the R was only 3%). Accordingly, ANFIS was then further used to develop gender-based models.

#### 3.2.3. Finding the Representative Region of CVL at One Year as the Outcome

To find the most representative outcome between the 12 regions, the performance of each cartilage region was examined with the ANFIS methodology using the ten variables. Data showed (Table 2) that the medial condyle and the global tibial plateau had the highest accuracy. Moreover, although the R value was identical for these two regions, the RMSE and MAE were lower in the medial condyle. Therefore, the medial condyle region was elected as the most representative outcome.

#### 3.2.4. Uncovering the Most Effective Input Variable (Risk Factors and BC Regions) Combination

Further, by using the medial condyle region as the representative outcome, the systematic controllability variable reduction was employed to find the optimal input combination for prediction, i.e., the best statistical indices and the lowest number of variables possible.

As the first step, 55 different variable combinations (Table 3) were analyzed, in which the M1 represented the model with all ten BC region variables, and M2-M55 corresponded to all possible combinations for nine (M2-11) and eight (M12-M55) variables.

As shown in Table 3, the lack of one of the variables provided in M1 resulted in a decrease in accuracy (R, RMSE, MAE) in all models, but in M11 (missing age), in which the accuracy was about the same as in M1. Therefore, age can be considered as a potential variable that can be removed with the least impact on the accuracy of the ML model in outcome prediction. Further investigation with eight variables revealed that among the models, M20 (missing age and medial tibial plateau) performed as M1 and better than M11. Thus, the combination of age and medial tibial plateau can be removed from the input variables without any impact on the accuracy of outcome prediction.

Therefore, by using M20, seven and six variables were analyzed. The results (Table 4) for models with seven variables revealed that M20-8 (missing age, medial tibial plateau, and BMI) outperformed not only other combinations but also M20. When looking at six variables, M20-13 (missing age, lateral central condyle, medial posterior condyle, and medial tibial plateau) presents statistical indices similar to M1 and M20, but the R has a 1% difference with M20-8.

Further, with M20-13, we looked at the five variables (Table 5). The model M20-13-6 with five variables (in addition to the missing M20-13 variables, BMI is also lacking) had equal statistical indices to M1. ML models with a lower number of variables were also assessed and data demonstrated a significantly reduced accuracy (data not shown). Consequently, the best prediction model for medial condyle CVL at one year was M20-13-6, which employed only five knee BC regions at baseline including lateral and medial trochlea, lateral posterior condyle, lateral tibial plateau, and medial central condyle.

**Figure 2:**
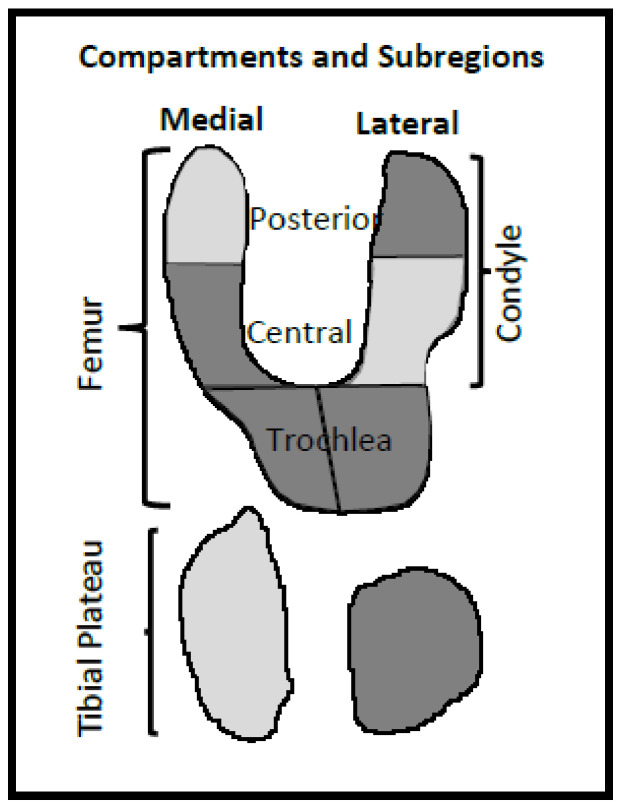
Representation of the knee bone curvature regions for the best prediction model for medial condyle cartilage volume loss at one year. The regions represented with the model M20-13-6 (dark) are lateral and medial trochlea, lateral posterior condyle, lateral tibial plateau, and medial central condyle.

Figure 2 shows a representation of the knee with the subregions in which the M20-13-6 variable combination is denoted (dark regions).

Table 6 recapitulates the obtained results of the proposed systematic controllability feature reduction for the prediction of medial condyle CVL at one year. Discrimination of the model M20-13-6 for each gender (Table 7) showed that the man has slightly better statistical indices than the woman.

#### 3.2.5. Impact of Each M20-13-6 Variable in Medial Condyle Volume Loss at One Year Forecasting

Table 8 shows the statistical indices of M20-13-6, wherein the effect of each feature was assessed by removing one variable at a time (M20-13-6-1 to M20-13-6-5). Data revealed that the lateral tibial plateau (M20-13-6-3), followed by the medial central condyle (M20-13-6-1), have a higher impact on the outcome forecasting; the worst statistical values were obtained when they were excluded. A lower impact (i.e., best statistical indices) was achieved with the lateral posterior condyle and the lateral and medial trochlea, respectively.

#### 3.2.6. Performance of the M-20-13-6 Model on All 12 CVL Region Outcomes

Next, we assessed the predictive validity of the selected ML algorithm on the other 11 cartilage regions (Table 9). Data showed very good accuracy for both genders and all 12 cartilage regions in the testing stage. The lowest accuracy in men was for the medial tibial plateau (R, 0.82; RMSE, 0.045; MAE, 0.030) and women, the medial femur (R, 0.79; RMSE, 0.027; MAE, 1.019) both in the testing stage. These results demonstrate the high performance of the M20-13-6 algorithm in the prediction of CVL in all 12 studied regions at one year based on five BC regions at the baseline.

### 3.3. Validation of the Developed ML Model with an External Cohort from a Clinical Trial

The purpose of a ML-based predictive model is to offer valid outcome predictions for new individuals that assure the generalizability of the model. To this end, the performance of the M20-13-6 model was evaluated using an external cohort (Naproxen) on all 12 cartilage regions studied discriminating men and women. The predictive model (Table 10) demonstrated very good accuracy for men and women (R ≥ 0.78), except for the medial tibial plateau for women.

## 4. Discussion

At present, we cannot discriminate, early during the OA process, patients for whom cartilage will degrade rapidly from those for whom the progression will be slow. Such discrimination would not only assist to modify the disease trajectory with a personalized clinical treatment plan but would represent a unique opportunity to intervene before cartilage degradation becomes too severe. Moreover, it would also enable patient screening for clinical trials for the development of DMOADs. Indeed, such drug trials have not yet achieved significant results, which appears to be mainly due to OA recruitment, in that patients have, for the most part, moderate to severe cartilage damage. Consequently, the effect of a DMOAD could not be observed with enough statistical power. This study was undertaken to fulfill these needs.

To achieve CVL forecasting, evidence points toward the use of joint tissue markers and, more recently, BC was suggested for the knee. We developed a gender-based model in which five BC regions at baseline (lateral tibial plateau, medial central condyle, lateral posterior condyle, and lateral and medial trochlea) enable the prediction of 12 global and regional CVL at one year with very good accuracy for both genders: OAI, R ≥ 0.79 (testing stage) and Naproxen (validation) R ≥ 0.78, except for the medial tibial plateau for women.

As we aimed to detect CVL for multiple (12 global/regional) outcomes, a two-phase ML-based methodology was performed. In Phase 1, after comparing the accuracy and benefits of five ML algorithms, ANFIS was found to be the most reliable for prediction. The selection of ANFIS was not surprising as it has the advantage over other ML methodologies of capturing the nonlinear structure of a problem, an adaptive capability and a rapid learning capacity as it combines a neural network with fuzzy logic, in addition to a significant potential for predicting systems with high uncertainty and in a dynamic nature.

Next, data showed that the most representative region of CVL (outcome) was the medial condyle. Such a finding could reflect that the medial tibiofemoral compartment of the knee, more specifically the medial condyle, displays a higher rate of cartilage change with greater sensitivity than the other regions [42,43,44,45], as well as being highly related to OA progression and total knee replacement [46,47,48].

In Phase 2, the relevant variables were selected. Reducing the number of variables for ML development and application saves resources. Moreover, having fewer misleading features not only improves the accuracy of a ML model but also removes multicollinearity, thus reducing the possibility of overfitting. To this end, we employed a systematic controllability variable reduction (removing the lowest cost features among all input variables) to identify the relevant ones.

In this study, of the five selected BC variables, the lateral tibial plateau and medial central condyle demonstrated the highest impact in prediction forecasting. This finding contrasts with a previous one in which two other BC regions, namely, medial posterior condyle and lateral central condyle, were found to be the best regions to predict CVL at two years [20]. In the current study, the weight of these two regions appeared to be somewhat important as they were eliminated only when eight variables were examined (M30). Removing these two variables resulted in a decrease of 10.5% in R, and an increase of about 27% in RMSE and MAE, compared to model M1 (all ten variables). It should also be taken into consideration that the period examined between the two studies, as well as the methodology varied, which could be responsible for the change in the selected variables.

Here, the selection of the lateral tibial plateau and medial central condyle was not unexpected as they both showed a high level of bony remodeling during OA. Indeed, the tibial plateau demonstrated expansion and increased depression during the OA process [49,50,51,52], and bony changes in the lateral tibial plateau were associated with the presence of radiographic OA [53]. Moreover, uneven lateral support of the tibial plateau has been reported to be a key factor that leads to the non-uniform settlement of the knee and a shift of the mechanical axis to the medial compartment, more specifically, on the medial central condyle [54]. The stresses engendered could be responsible for the reported flattening of the medial central condyle bone during OA [14,23]. Bone remodeling in the medial central condyle could also be due to the presence of a high level of BMLs in the OA knee in this region [55]. Although BML was removed from our BC segmentation [27], such subchondral bone changes are suggested to increase the levels of contact stresses, thus, bone remodeling [56].

Even though all the 12 studied global and regional cartilage regions could be predicted with high accuracy with the OAI participants, validation using OA patients from a clinical trial (Naproxen) showed that generalization was attained in all cartilage regions, except in the medial tibial plateau for women. The lower accuracy in this region in women could reflect the fact that (i) compared to the OAI, participants from the Naproxen cohort displayed more disease severity, as ascertained by the clinical parameters, (ii) during the OA process, there was a high level of cartilage thinning/loss as well as inter-subject variability in this region [33,43,57], in addition to (iii) the cartilage volume of women being smaller than in men [58].

The finding that BMI was not included in the model with five variables was rather surprising as a link between BMI and knee bone remodeling has been previously reported [59]. However, this is still under debate as other studies have not shown such an association [53]. Of note, the weight of this variable was, to some extent, important as it was included when six variables were investigated.

Some challenges and limitations of this study should be acknowledged. First, ANFIS was selected as the best ML algorithm for model development. Because of the use of ten input variables, a limitation of this method could have been the high computational expense due to the high number of iterations needed to achieve high accuracy. However, care was given to the selection of the appropriate number and shape of membership function in the ANFIS model as they impact the accuracy of the final results and computational complexity of the ANFIS-based model, and although a challenging task, we were able to define the appropriate membership function, i.e., Gaussian (Table S1) for this study.

Second, in practice, for a given ML problem, multiple equivalent solutions in variable selections can exist [60]. A shortcoming of some variable selection methods is that they injudiciously identify only a single solution, minimizing a loss function like mean squared error, classification error, etc. Yet, a single solution is not proper when variable selections can be considered both for building a predictive model with high accuracy and for knowledge discovery. In this study, we opted to employ the systematic controllability variable reduction as, instead of giving only one solution, it deals with achieving the highest accuracy by removing the lowest cost variables. In addition, with this step-by-step variable reduction, not only the sensitivity but also the synergy between two variables for the estimation of the outcome could be evaluated.

Third, we could have used the cartilage volume as the outcome. We favored this tissue volume loss as to whether baseline cartilage volume predicts future cartilage loss is questionable.

Fourth, another challenge was the CVL period to be analyzed. We chose one year to ensure both a reliable assessment of cartilage change sensitivity and high patient retention for its use in clinical practice. However, a longer period was not evaluated, and the five input variables found could differ. The next step will be to explore, for a longer observation period, whether the developed ML model using the same BC variables could also predict with high accuracy CVL for all the studied regions. Although the purpose of this study was to evaluate BC as prognosis for CVL over time, assessing a longer period of cartilage loss (e.g., two to four years) could educate us on the collinearity between these two structures. In addition, validating the ML model for longer periods converts it into an application that can be of broader use in clinical practice.

This study has several strengths. The use of MRI to assess BC and cartilage volume at baseline permits for automation of these two knee structures (thus, avoiding human error) and quantitative segmentation/measurement in the same knee [27,32]. In addition, the 3D nature of the MRI data over radiographs for knee tissue measurement avoids difficulties in interpreting findings that may be related to positioning during image acquisition and to projection effects. Moreover, when studying BC in OA, care should be taken not to confuse the osteophytes and BMLs with true differences in the bone. This putative problem was circumvented by the exclusion of these two tissues in the measurement methodology used [27]. Finally, special emphasis needs to be given to the validation (reproducibility) of data using an external clinical trial cohort which, in addition to mimicking patients seen in clinical routine, adds to the robustness and generalization of the developed ML model in that the accuracy persisted.

## 5. Conclusions

In this comprehensive study, we developed, for the first time, a reliable and generalizable ML model to predict global and regional CVL at one year based on five BC regions at the baseline, including the lateral tibial plateau, medial central condyle, lateral posterior condyle, and lateral and medial trochlea. This study offers a novel automated system for forecasting knee OA cartilage degradation as an important step toward OA precision medicine, which will significantly improve clinical prognosis with real-time patient monitoring.

## Figures and Tables

**Table 1:** Baseline participant characteristics.

	OAI	Naproxen	*p*-Value
	(*n* = 1246)	(*n* = 53)	
Gender, female—*n* (%)	659 (52.9)	33 (62.3)	0.180
Age—years	64.0 (56.0; 71.0)	63.0 (58.0; 66.0)	0.158
Body mass index—kg/m^2^	29.1 (26.2; 32.6)	31.5 (27.5; 34.9)	**0.009**
Target knee—*n* (%)	(*n* = 1245)		0.256
Right	732 (58.8)	27 (50.9)	
Left	513 (41.2)	26 (49.1)	
WOMAC			
	(*n* = 1245)		
Pain score (0–20)	3.0 (0.0; 6.0)	10.8 (9.8; 12.6)	**<0.0001**
	(*n* = 1240)		
Function score (0–68)	7.0 (1.0; 18.2)	35.4 (30.6; 46.0)	**<0.0001**
	(*n* = 1243)		
Stiffness score (0–8)	2.0 (0.0; 3.0)	5.2 (3.8; 6.2)	**<0.0001**
	(*n* = 1238)		
Total score (0–96)	11.8 (3.0; 27.0)	51.4 (44.9; 64.2)	**<0.0001**
Kellgren–Lawrence grade—*n* (%)	(*n* = 1245)		
0	69 (5.6)	Not available	
1	102 (8.2)		
2	375 (30.1)		
3	528 (42.4)		
4	171 (13.7)		
Joint space width, mm	3.1 (1.9; 3.7)	3.7 (2.5; 4.2)	**0.003**
Cartilage volume at baseline, mm^3^			
Global knee	11,843 (9867; 14,898)	10,649 (9272; 12,628)	**0.024**
Lateral compartment	6662 (5465; 8147)	5811 (4891; 6931)	**0.003**
Medial compartment	5266 (4222; 6761)	4901 (4501; 5760)	0.344
Bone curvature, m^−1^			
Global knee	27.1 (23.9; 30.1)	28.0 (24.9; 31.3)	0.306
Lateral compartment	29.9 (26.3; 33.0)	30.0 (24.8; 34.6)	0.974
Medial compartment	23.9 (20.3; 27.9)	26.0 (21.3; 30.2)	**0.028**

Data are from the Osteoarthritis Initiative (OAI) or the Naproxen arm of the Licofelone clinical trial cohorts [28]. Data are median (1st quartile; 3rd quartile) unless otherwise indicated. Continuous variables were compared using the Wilcoxon Mann–Whitney test and proportions using the Pearson Chi-Square test. *p* values < 0.050 (in bold) were considered statistically different. *n*, number of participants; WOMAC, Western Ontario and McMaster Universities Osteoarthritis Index.

**Table 2:** Performance of prediction of cartilage volume loss at one year in 12 regions using the ANFIS methodology.

Outcome	R	RMSE	MAE
Global knee	0.78	0.020	0.015
Global femur	0.74	0.022	0.015
Global condyles	0.81	0.021	0.016
Global tibial plateau	**0.86**	**0.027**	**0.020**
Lateral compartment	0.78	0.021	0.015
Lateral femur	0.77	0.023	0.016
Lateral condyle	0.78	0.025	0.017
Lateral tibial plateau	0.74	0.036	0.026
Medial compartment	0.77	0.027	0.020
Medial femur	0.78	0.026	0.018
Medial condyle	**0.86**	**0.026**	**0.018**
Medial tibial plateau	0.76	0.057	0.040

R, correlation coefficient; RMSE, root mean square error; MAE, mean absolute error. Bold indicates data of the most representative regions.

**Table 3:** Models in the systematic controllability variable reduction approach for nine and eight bone curvature region variables with their performance in predicting medial condyle cartilage volume loss at one year.

Ten Variables	Model	In1	In2	In3	In4	In5	In6	In7	In8	In9	In10	R	RMSE	MAE
	M1	•	•	•	•	•	•	•	•	•	•	0.86	0.026	0.018
**Combinations with nine variables**	M2	•	•	•	•	•	•	•	•	•		0.75	0.034	0.024
	M3	•	•	•	•	•	•	•	•		•	0.81	0.030	0.021
	M4	•	•	•	•	•	•	•		•	•	0.72	0.036	0.024
	M5	•	•	•	•	•	•		•	•	•	0.77	0.033	0.023
	M6	•	•	•	•	•		•	•	•	•	0.75	0.034	0.024
	M7	•	•	•	•		•	•	•	•	•	0.78	0.033	0.024
	M8	•	•	•		•	•	•	•	•	•	0.80	0.031	0.022
	M9	•	•		•	•	•	•	•	•	•	0.80	0.031	0.022
	M10	•		•	•	•	•	•	•	•	•	0.78	0.032	0.023
	M11		•	•	•	•	•	•	•	•	•	0.85	0.028	0.019
**Input combinations with eight variables**	M12	•	•	•	•	•	•	•	•			0.77	0.033	0.023
	M13	•	•	•	•	•	•	•		•		0.81	0.030	0.022
	M14	•	•	•	•	•	•		•	•		0.79	0.031	0.022
	M15	•	•	•	•	•		•	•	•		0.76	0.034	0.024
	M16	•	•	•	•		•	•	•	•		0.76	0.034	0.025
	M17	•	•	•		•	•	•	•	•		0.73	0.035	0.026
	M18	•	•		•	•	•	•	•	•		0.76	0.033	0.023
	M19	•		•	•	•	•	•	•	•		0.72	0.036	0.026
	**M20**		•	•	•	•	•	•	•	•		**0.86**	**0.026**	**0.018**
	M21	•	•	•	•	•	•	•			•	0.81	0.031	0.021
	M22	•	•	•	•	•	•		•		•	0.73	0.036	0.025
	M23	•	•	•	•	•		•	•		•	0.79	0.032	0.022
	M24	•	•	•	•		•	•	•		•	0.83	0.029	0.020
	M25	•	•	•		•	•	•	•		•	0.81	0.030	0.021
	M26	•	•		•	•	•	•	•		•	0.76	0.034	0.024
	M27	•		•	•	•	•	•	•		•	0.83	0.029	0.021
	M28		•	•	•	•	•	•	•		•	0.85	0.028	0.019
	M29	•	•	•	•	•	•			•	•	0.76	0.033	0.025
	M30	•	•	•	•	•		•		•	•	0.77	0.033	0.023
	M31	•	•	•	•		•	•		•	•	0.77	0.033	0.024
	M32	•	•	•		•	•	•		•	•	0.80	0.031	0.022
	M33	•	•		•	•	•	•		•	•	0.77	0.033	0.024
	M34	•		•	•	•	•	•		•	•	0.70	0.037	0.028
	M35		•	•	•	•	•	•		•	•	0.80	0.031	0.021
	M36	•	•	•	•	•			•	•	•	0.76	0.034	0.025
	M37	•	•	•	•		•		•	•	•	0.83	0.029	0.021
	M38	•	•	•		•	•		•	•	•	0.82	0.030	0.021
	M39	•	•		•	•	•		•	•	•	0.83	0.029	0.020
	M40	•		•	•	•	•		•	•	•	0.82	0.030	0.021
	M41		•	•	•	•	•		•	•	•	0.83	0.029	0.019
	M42	•	•	•	•			•	•	•	•	0.77	0.033	0.024
	M43	•	•	•		•		•	•	•	•	0.80	0.031	0.022
	M44	•	•		•	•		•	•	•	•	0.79	0.032	0.023
	M45	•		•	•	•		•	•	•	•	0.81	0.030	0.021
	M46		•	•	•	•		•	•	•	•	0.82	0.030	0.021
	M47	•	•	•			•	•	•	•	•	0.81	0.030	0.021
	M48	•	•		•		•	•	•	•	•	0.77	0.033	0.024
	M49	•		•	•		•	•	•	•	•	0.81	0.030	0.021
	M50		•				•	•	•	•	•	0.83	0.029	0.020
	M51	•		•		•	•	•	•	•	•	0.83	0.029	0.019
	M52		•	•		•	•	•	•	•	•	0.82	0.030	0.022
	M53	•			•	•	•	•	•	•	•	0.79	0.032	0.021
	M54		•		•	•	•	•	•	•	•	0.83	0.029	0.020
	M55			•	•	•	•	•	•	•	•	0.69	0.037	0.026

The symbol • indicates the presence of the bone curvature region variables in the model. In1, Age; In2, Body mass index; In3, Lateral trochlea; In4, Lateral central condyle; In5, Lateral posterior condyle; In6, Lateral tibial plateau; In7, Medial trochlea; In8, Medial central condyle; In9, Medial posterior condyle; In10, Medial tibial plateau. Model (M)1 includes all the ten bone curvature region variables; M2 to M11, nine variables, M12 to M55, eight variables. The horizontal bar indicates the separation between the variable combinations. R, correlation coefficient; RMSE, root mean square error; MAE, mean absolute error.

**Table 4:** Models in the systematic controllability variable reduction approach for seven and six bone curvature region variables with their performance in predicting medial condyle cartilage volume loss at one year.

Ten Variables	Model	In1	In2	In3	In4	In5	In6	In7	In8	In9	In10	R	RMSE	MAE
	M1	•	•	•	•	•	•	•	•	•	•	0.86	0.026	0.018
**Combinations with seven variables**	M20-1		•	•	•	•	•	•	•			0.80	0.031	0.022
	M20-2		•	•	•	•	•	•		•		0.83	0.029	0.021
	M20-3		•	•	•	•	•		•	•		0.84	0.029	0.020
	M20-4		•	•	•	•		•	•	•		0.82	0.030	0.022
	M20-5		•	•	•		•	•	•	•		0.79	0.032	0.023
	M20-6		•	•		•	•	•	•	•		0.81	0.030	0.021
	M20-7		•		•	•	•	•	•	•		0.82	0.030	0.020
	**M20-8**			•	•	•	•	•	•	•		**0.87**	**0.026**	**0.018**
**Combinations with six variables**	M20-9		•	•	•	•	•	•				0.82	0.029	0.021
	M20-10		•	•	•	•	•		•			0.85	0.028	0.019
	M20-11		•	•	•	•		•	•			0.76	0.034	0.023
	M20-12		•	•	•		•	•	•			0.83	0.029	0.020
	**M20-13**		•	•		•	•	•	•			**0.86**	**0.026**	**0.018**
	M20-14		•		•	•	•	•	•			0.80	0.031	0.021
	M20-15			•	•	•	•	•	•			0.82	0.030	0.021
	M20-16		•	•	•	•	•			•		0.78	0.033	0.024
	M20-17		•	•	•	•		•		•		0.79	0.032	0.023
	M20-18		•	•	•		•	•		•		0.78	0.033	0.023
	M20-19		•	•		•	•	•		•		0.79	0.032	0.022
	M20-20		•		•	•	•	•		•		0.72	0.036	0.026
	M20-21			•	•	•	•	•		•		0.68	0.038	0.027
	M20-22		•	•	•	•			•	•		0.85	0.027	0.019
	M20-23		•	•	•		•		•	•		0.79	0.032	0.022
	M20-24		•	•		•	•		•	•		0.84	0.028	0.021
	M20-25		•		•	•	•		•	•		0.82	0.030	0.021
	M20-26			•	•	•	•		•	•		0.68	0.038	0.027
	M20-27		•	•	•			•	•	•		0.78	0.032	0.023
	M20-28		•	•		•		•	•	•		0.81	0.030	0.021
	M20-29		•			•		•	•	•		0.66	0.039	0.028
	M20-30			•	•	•		•	•	•		0.72	0.036	0.026
	M20-31		•	•			•	•	•	•		0.81	0.030	0.022
	M20-32		•		•		•	•	•	•		0.71	0.037	0.026
	M20-33			•	•		•	•	•	•		0.72	0.036	0.025
	M20-34		•			•	•	•	•	•		0.84	0.028	0.019
	M20-35			•		•	•	•	•	•		0.73	0.035	0.026
	M20-36				•	•	•	•	•	•		0.71	0.037	0.026

The symbol • indicates the presence of the bone curvature region variable in the model. In1, Age; In2, Body mass index (BMI); In3, Lateral trochlea; In4, Lateral central condyle, In5, Lateral posterior condyle, In6, Lateral tibial plateau; In7, Medial trochlea; In8, Medial central condyle; In9, Medial posterior condyle; In10, Medial tibial plateau. Model (M)1 includes all the ten bone curvature region variables; M20-1 to M20-8, models with seven variables; M20-9 to M20-36, models with six variables. The horizontal bar indicates the separation between the variable combinations. R, correlation coefficient; RMSE, root mean square error; MAE, mean absolute error.

**Table 5:** Models in the systematic controllability variable reduction approach for five bone curvature region variables and performance in predicting medial condyle volume loss at one year.

Ten Variables	Model	In1	In2	In3	In4	In5	In6	In7	In8	In9	In10	R	RMSE	MAE
	M1	•	•	•	•	•	•	•	•	•	•	0.86	0.026	0.018
**Combinations with five variables**	M20-13-1		•	•		•	•	•				0.78	0.035	0.025
	M20-13-2		•	•		•	•		•			0.83	0.031	0.023
	M20-13-3		•	•		•		•	•			0.82	0.032	0.023
	M20-13-4		•	•			•	•	•			0.82	0.032	0.023
	M20-13-5		•			•	•	•	•			0.75	0.037	0.027
	M20-13-6			•		•	•	•	•			**0.86**	**0.026**	**0.018**

The symbol • indicates the presence of the bone curvature region variable in the model. In1, Age; In2, Body mass index; In3, Lateral trochlea; In4, Lateral central condyle; In5, Lateral posterior condyle; In6, Lateral tibial plateau; In7, Medial trochlea; In8, Medial central condyle; In9, Medial posterior condyle; In10, Medial tibial plateau. Model (M)1 includes all the ten bone curvature region variables; M20-13-1 to M20-13-6, models with five variables. R, correlation coefficient; RMSE, root mean square error; MAE, mean absolute error.

**Table 6:** The best models obtained with a decreasing number of bone curvature region variables.

Variable Number	Model	In1	In2	In3	In4	In5	In6	In7	In8	In9	In10	R	RMSE	MAE
**10**	M1	•	•	•	•	•	•	•	•	•	•	0.86	0.026	0.018
**9**	M11		•	•	•	•	•	•	•	•	•	0.85	0.028	0.019
**8**	M20		•	•	•	•	•	•	•	•		0.86	0.026	0.018
**7**	M20-8			•	•	•	•	•	•	•		0.87	0.026	0.018
**6**	M20-13		•	•		•	•	•	•			0.86	0.026	0.018
**5**	M20-13-6			•		•	•	•	•			0.86	0.026	0.018

The symbol • indicates the presence of the bone curvature region variable in the model. In1, Age; In2, Body mass index; In3, Lateral trochlea; In4, Lateral central condyle; In5, Lateral posterior condyle; In6, Lateral tibial plateau; In7, Medial trochlea; In8, Medial central condyle; In9, Medial posterior condyle; In10, Medial tibial plateau. Model (M)1 includes all the ten bone curvature region variables, M11, the best model with nine variables; M20, the best model with eight variables; M20-8, the best model with seven variables; M20-13, the best model with six variables; M20-13-6, the best model with five variables. R, correlation coefficient; RMSE, root mean square error; MAE, mean absolute error.

**Table 7:** The best selected model in which the gender was discriminated.

Model	Man			Woman		
	R	RMSE	MAE	R	RMSE	MAE
M20-13-6	0.90	0.023	0.016	0.84	0.029	0.020

Model (M)20-13-6 with five bone curvature variables. R, correlation coefficient; RMSE, root mean square error; MAE, mean absolute error.

**Table 8:** Impact of each M20-13-6 bone curvature region variable in medial condyle cartilage volume loss at one year forecasting.

Model	In1	In2	In3	In4	In5	In6	In7	In8	In9	In10	R	RMSE	MAE
M20-13-6			•		•	•	•	•			0.86	0.026	0.018
M20-13-6-1			•		•	•	•				0.76	0.025	0.034
M20-13-6-2			•		•	•		•			0.79	0.024	0.032
M20-13-6-3			•		•		•	•			0.74	0.026	0.035
M20-13-6-4			•			•	•	•			0.81	0.022	0.031
M20-13-6-5					•	•	•	•			0.81	0.023	0.031

The symbol • indicates the presence of the bone curvature region variable in the model. In1, Age; In2, Body mass index; In3, Lateral trochlea; In4, Lateral central condyle; In5, Lateral posterior condyle; In6, Lateral tibial plateau; In7, Medial trochlea; In8, Medial central condyle; In9, Medial posterior condyle; In10, Medial tibial plateau. Model (M)20-13-6, the best model with five bone curvature region variables; M20-13-6-1 to M20-13-6-5, the model with four variables. R, correlation coefficient; RMSE, root mean square error; MAE, mean absolute error.

**Table 9:** Performance of prediction of cartilage volume loss at one year in 12 cartilage regions using five bone curvature regions at the baseline.

Outcome	Man						Woman					
	Training Stage			Testing Stage			Training Stage			Testing Stage		
	R	RMSE	MAE	R	RMSE	MAE	R	RMSE	MAE	R	RMSE	MAE
Global knee	0.87	0.013	0.010	0.87	0.016	0.012	0.87	0.018	0.013	0.85	0.017	0.013
Global femur	0.85	0.014	0.010	0.92	0.014	0.011	0.86	0.018	0.013	0.86	0.017	0.012
Global condyle	0.86	0.015	0.012	0.90	0.018	0.012	0.82	0.023	0.016	0.85	0.020	0.015
Global tibial Plateau	0.85	0.023	0.015	0.88	0.020	0.014	0.87	0.029	0.020	0.85	0.027	0.018
Lateral compartment	0.88	0.014	0.010	0.91	0.014	0.010	0.84	0.021	0.015	0.81	0.019	0.015
Lateral femur	0.89	0.014	0.010	0.91	0.015	0.011	0.86	0.020	0.014	0.86	0.018	0.012
Lateral condyle	0.89	0.016	0.012	0.89	0.020	0.014	0.84	0.023	0.016	0.88	0.019	0.014
Lateral tibial plateau	0.85	0.023	0.017	0.88	0.023	0.018	0.88	0.031	0.021	0.87	0.026	0.018
Medial compartment	0.88	0.019	0.014	0.92	0.016	0.012	0.85	0.023	0.016	0.85	0.023	0.016
Medial femur	0.85	0.020	0.014	0.88	0.020	0.015	0.83	0.025	0.017	0.79	0.027	0.019
Medial condyle	0.90	0.022	0.015	0.90	0.023	0.016	0.83	0.029	0.021	0.84	0.029	0.020
Medial tibial plateau	0.89	0.041	0.030	0.82	0.045	0.030	0.82	0.052	0.036	0.80	0.051	0.033

The model was developed using individuals from the Osteoarthritis Initiative (OAI) cohort. R, correlation coefficient; RMSE, root mean square error; MAE, mean absolute error.

**Table 10:** Validation of the M20-13-6 model in the prediction of cartilage volume loss at one year in 12 cartilage regions using five bone curvature regions at the baseline.

Outcome	Man			Woman		
	R	RMSE	MAE	R	RMSE	MAE
Global knee	0.89	0.018	0.015	0.89	0.022	0.016
Global femur	0.90	0.019	0.015	0.84	0.026	0.017
Global condyle	0.85	0.023	0.018	0.81	0.032	0.019
Global tibial Plateau	0.88	0.027	0.020	0.79	0.032	0.028
Lateral compartment	0.94	0.021	0.016	0.86	0.022	0.017
Lateral femur	0.92	0.021	0.016	0.83	0.027	0.018
Lateral condyle	0.96	0.026	0.018	0.86	0.031	0.020
Lateral tibial plateau	0.94	0.040	0.023	0.87	0.028	0.023
Medial compartment	0.87	0.027	0.021	0.90	0.031	0.023
Medial femur	0.88	0.027	0.022	0.89	0.030	0.021
Medial condyle	0.91	0.026	0.021	0.87	0.037	0.027
Medial tibial plateau	0.78	0.041	0.029	*0.47*	*0.062*	*0.045*

The model was validated using osteoarthritis patients from a clinical trial cohort (Naproxen). R, correlation coefficient; RMSE, root mean square error; MAE, mean absolute error.

## Data Availability

Data from the Osteoarthritis Initiative (OAI) cohort used in this study are publicly available (https://data-archive.nimh.nih.gov/oai/, last accessed date: 25 October 2019). The additional data used and/or analyzed for the current study are available from the corresponding author upon reasonable request, as long as the request is evaluated as scientifically relevant.

## Supplementary Materials

The following supporting information can be downloaded at: https://www.mdpi.com/article/10.3390/biomedicines10061247/s1, Table S1: A machine learning model to predict knee osteoarthritis cartilage volume changes over time using baseline bone curvature; Table S2: Performance of five machine learning algorithms in predicting cartilage volume loss at one year in 12 regions.

## References

[1] GBD 2015 Disease and Injury Incidence and Prevalence Collaborators (2016). Global, regional, and national incidence, prevalence, and years lived with disability for 310 diseases and injuries, 1990–2015: A systematic analysis for the Global Burden of Disease Study 2015. Lancet.

[2] GBD Disease and Injury Incidence and Prevalence Collaborators (2018). Global, regional, and national incidence, prevalence, and years lived with disability for 354 diseases and injuries for 195 countries and territories, 1990–2017: A systematic analysis for the Global Burden of Disease Study 2017. Lancet.

[3] Martel-Pelletier J., Barr A.J., Cicuttini F.M., Conaghan P.G., Cooper C., Goldring M.B., Goldring S.R., Jones G., Teichtahl A.J., Pelletier J.P. (2016). Osteoarthritis. Nat. Rev. Dis. Primers.

[4] Amoako A.O., Pujalte G.G. (2014). Osteoarthritis in young, active, and athletic individuals. Clin. Med. Insights Arthritis Musculoskelet. Disord..

[5] Roos E.M., Arden N.K. (2016). Strategies for the prevention of knee osteoarthritis. Nat. Rev. Rheumatol..

[6] Vina E.R., Kwoh C.K. (2018). Epidemiology of osteoarthritis: Literature update. Curr. Opin. Rheumatol..

[7] Solomon D.H., Rassen J.A., Glynn R.J., Lee J., Levin R., Schneeweiss S. (2010). The comparative safety of analgesics in older adults with arthritis. Arch. Intern. Med..

[8] FDA Drug Safety Communication: FDA Strengthens Warning That Non-Aspirin Nonsteroidal Anti-Inflammatory Drugs (NSAIDs) Can Cause Heart Attacks or Strokes (7-9-2015). https://www.fda.gov/Drugs/DrugSafety/ucm451800.htm.

[9] Teichtahl A.J., Wluka A.E., Davies-Tuck M.L., Cicuttini F.M. (2008). Imaging of knee osteoarthritis. Best Pract. Res. Clin. Rheumatol..

[10] Guermazi A., Roemer F.W., Burstein D., Hayashi D. (2011). Why radiography should no longer be considered a surrogate outcome measure for longitudinal assessment of cartilage in knee osteoarthritis. Arthritis Res. Ther..

[11] Lotz M., Martel-Pelletier J., Christiansen C., Brandi M.L., Bruyere O., Chapurlat R., Collette J., Cooper C., Giacovelli G., Kanis J.A. (2013). Value of biomarkers in osteoarthritis: Current status and perspectives. Ann. Rheum. Dis..

[12] Jones G., Ding C., Scott F., Glisson M., Cicuttini F. (2004). Early radiographic osteoarthritis is associated with substantial changes in cartilage volume and tibial bone surface area in both males and females. Osteoarthr. Cartil..

[13] Ding C., Cicuttini F., Jones G. (2007). Tibial subchondral bone size and knee cartilage defects: Relevance to knee osteoarthritis. Osteoarthr. Cartil..

[14] Neogi T., Bowes M.A., Niu J., De Souza K.M., Vincent G.R., Goggins J., Zhang Y., Felson D.T. (2013). Magnetic resonance imaging-based three-dimensional bone shape of the knee predicts onset of knee osteoarthritis: Data from the osteoarthritis initiative. Arthritis Rheum..

[15] Everhart J.S., Siston R.A., Flanigan D.C. (2014). Tibiofemoral subchondral surface ratio (SSR) is a predictor of osteoarthritis symptoms and radiographic progression: Data from the Osteoarthritis Initiative (OAI). Osteoarthr. Cartil..

[16] Hunter D., Nevitt M., Lynch J., Kraus V.B., Katz J.N., Collins J.E., Bowes M., Guermazi A., Roemer F.W., Losina E. (2016). Longitudinal validation of periarticular bone area and 3D shape as biomarkers for knee OA progression? Data from the FNIH OA Biomarkers Consortium. Ann. Rheum. Dis..

[17] Wise B.L., Niu J., Zhang Y., Liu F., Pang J., Lynch J.A., Lane N.E. (2018). Bone shape mediates the relationship between sex and incident knee osteoarthritis. BMC Musculoskelet. Disord..

[18] Hohe J., Ateshian G., Reiser M., Englmeier K.H., Eckstein F. (2002). Surface size, curvature analysis, and assessment of knee joint incongruity with MRI in vivo. Magn. Reson. Med..

[19] Wise B.L., Liu F., Kritikos L., Lynch J.A., Parimi N., Zhang Y., Lane N.E. (2016). The association of distal femur and proximal tibia shape with sex: The Osteoarthritis Initiative. Semin. Arthritis Rheum..

[20] Raynauld J.P., Pelletier J.P., Delorme P., Dodin P., Abram F., Martel-Pelletier J. (2017). Bone curvature changes can predict the impact of treatment on cartilage volume loss in knee osteoarthritis: Data from a 2-year clinical trial. Rheumatology.

[21] Reichenbach S., Guermazi A., Niu J., Neogi T., Hunter D.J., Roemer F.W., McLennan C.E., Hernandez-Molina G., Felson D.T. (2008). Prevalence of bone attrition on knee radiographs and MRI in a community-based cohort. Osteoarthr. Cartil..

[22] Wise B.L., Kritikos L., Lynch J.A., Liu F., Parimi N., Tileston K.L., Nevitt M.C., Lane N.E. (2014). Proximal femur shape differs between subjects with lateral and medial knee osteoarthritis and controls: The Osteoarthritis Initiative. Osteoarthr. Cartil..

[23] Bowes M.A., Vincent G.R., Wolstenholme C.B., Conaghan P.G. (2015). A novel method for bone area measurement provides new insights into osteoarthritis and its progression. Ann. Rheum. Dis..

[24] Barr A.J., Dube B., Hensor E.M., Kingsbury S.R., Peat G., Bowes M.A., Sharples L.D., Conaghan P.G. (2016). The relationship between three-dimensional knee MRI bone shape and total knee replacement-a case control study: Data from the Osteoarthritis Initiative. Rheumatology.

[25] Bowes M.A., Kacena K., Alabas O.A., Brett A.D., Dube B., Bodick N., Conaghan P.G. (2020). Machine-learning, MRI bone shape and important clinical outcomes in osteoarthritis: Data from the Osteoarthritis Initiative. Ann. Rheum. Dis..

[26] Chang G.H., Park L.K., Le N.A., Jhun R.S., Surendran T., Lai J., Seo H., Promchotichai N., Yoon G., Scalera J. (2021). Subchondral bone length in knee osteoarthritis: A deep learning derived imaging measure and its association with radiographic and clinical outcomes. Arthritis Rheumatol..

[27] Dodin P., Martel-Pelletier J., Pelletier J.P., Abram F. (2011). A fully automated human knee 3D MRI bone segmentation using the ray casting technique. Med. Biol. Eng. Comput..

[28] Raynauld J.P., Martel-Pelletier J., Bias P., Laufer S., Haraoui B., Choquette D., Beaulieu A.D., Abram F., Dorais M., Vignon E. (2009). Protective effects of licofelone, a 5-lipoxygenase and cyclo-oxygenase inhibitor, versus naproxen on cartilage loss in knee osteoarthritis: A first multicentre clinical trial using quantitative MRI. Ann. Rheum. Dis..

[29] Jamshidi A., Leclercq M., Labbe A., Pelletier J.P., Abram F., Droit A., Martel-Pelletier J. (2020). Identification of the most important features of knee osteoarthritis structural progressors using machine learning methods. Ther. Adv. Musculoskelet. Dis..

[30] Altman R.D., Gold G.E. (2007). Atlas of individual radiographic features in osteoarthritis, revised. Osteoarthr. Cartil..

[31] Bonakdari H., Jamshidi A., Pelletier J.P., Abram F., Tardif G., Martel-Pelletier J. (2021). A warning machine learning algorithm for early knee osteoarthritis structural progressor patient screening. Ther. Adv. Musculoskel. Dis..

[32] Dodin P., Pelletier J.P., Martel-Pelletier J., Abram F. (2010). Automatic human knee cartilage segmentation from 3D magnetic resonance images. IEEE Trans. Biomed. Eng..

[33] Martel-Pelletier J., Roubille C., Abram F., Hochberg M.C., Dorais M., Delorme P., Raynauld J.P., Pelletier J.P. (2015). First-line analysis of the effects of treatment on progression of structural changes in knee osteoarthritis over 24 months: Data from the osteoarthritis initiative progression cohort. Ann. Rheum. Dis..

[34] Raynauld J.P., Martel-Pelletier J., Berthiaume M.J., Labonté F., Beaudoin G., de Guise J.A., Bloch D.A., Choquette D., Haraoui B., Altman R.D. (2004). Quantitative magnetic resonance imaging evaluation of knee osteoarthritis progression over two years and correlation with clinical symptoms and radiologic changes. Arthritis Rheum..

[35] Kauffmann C., Gravel P., Godbout B., Gravel A., Beaudoin G., Raynauld J.P., Martel-Pelletier J., Pelletier J.P., de Guise J.A. (2003). Computer-aided method for quantification of cartilage thickness and volume changes using MRI: Validation study using a synthetic model. IEEE Trans. Biomed. Eng..

[36] Raynauld J.P., Kauffmann C., Beaudoin G., Berthiaume M.J., de Guise J.A., Bloch D.A., Camacho F., Godbout B., Altman R.D., Hochberg M. (2003). Reliability of a quantification imaging system using magnetic resonance images to measure cartilage thickness and volume in human normal and osteoarthritic knees. Osteoarthr. Cartil..

[37] Breiman L. (2001). Random forests. Mach. Learn..

[38] Quinlan J.R. Learning with Continuous Classes. Proceedings of the Proceedings from the 5th Australian Joint Conference on Artificial Intelligence.

[39] Rosenblatt F. (1962). Principles of Neurodynamics: Perceptrons and the Theory of Brian Mechanisms.

[40] Jang J.S.R. (1993). ANFIS: Adaptive-network-based fuzzy inference system. IEEE Trans. Syst. Man Cybern..

[41] D’Agostino R.B., Stephens M.A. (1986). Goodness-of-Fit. Techniques.

[42] Ledingham J., Regan M., Jones A., Doherty M. (1993). Radiographic patterns and associations of osteoarthritis of the knee in patients referred to hospital. Ann. Rheum. Dis..

[43] Pelletier J.P., Raynauld J.P., Berthiaume M.J., Abram F., Choquette D., Haraoui B., Beary J.F., Cline G.A., Meyer J.M., Martel-Pelletier J. (2007). Risk factors associated with the loss of cartilage volume on weight-bearing areas in knee osteoarthritis patients assessed by quantitative magnetic resonance imaging: A longitudinal study. Arthritis Res. Ther..

[44] Eckstein F., Nevitt M., Gimona A., Picha K., Lee J.H., Davies R.Y., Dreher D., Benichou O., Le Graverand M.P., Hudelmaier M. (2011). Rates of change and sensitivity to change in cartilage morphology in healthy knees and in knees with mild, moderate, and end-stage radiographic osteoarthritis: Results from 831 participants from the Osteoarthritis Initiative. Arthritis Care Res..

[45] Eckstein F., Kwoh C.K., Boudreau R.M., Wang Z., Hannon M.J., Cotofana S., Hudelmaier M.I., Wirth W., Guermazi A., Nevitt M.C. (2013). Quantitative MRI measures of cartilage predict knee replacement: A case-control study from the Osteoarthritis Initiative. Ann. Rheum. Dis..

[46] Pelletier J.P., Cooper C., Peterfy C., Reginster J.Y., Brandi M.L., Bruyere O., Chapurlat R., Cicuttini F., Conaghan P.G., Doherty M. (2013). What is the predictive value of MRI for the occurrence of knee replacement surgery in knee osteoarthritis?. Ann. Rheum. Dis..

[47] Eckstein F., Collins J.E., Nevitt M.C., Lynch J.A., Kraus V.B., Katz J.N., Losina E., Wirth W., Guermazi A., Roemer F.W. (2015). Brief report: Cartilage thickness change as an imaging biomarker of knee osteoarthritis progression: Data from the foundation for the National Institutes of Health Osteoarthritis Biomarkers Consortium. Arthritis Rheumatol..

[48] Dorio M., Hunter D.J., Collins J.E., Asher R., Eckstein F., Guermazi A., Roemer F.W., Deveza L.A. (2020). Association of baseline and change in tibial and femoral cartilage thickness and development of widespread full-thickness cartilage loss in knee osteoarthritis—Data from the Osteoarthritis Initiative. Osteoarthr. Cartil..

[49] Wang Y., Wluka A.E., Cicuttini F.M. (2005). The determinants of change in tibial plateau bone area in osteoarthritic knees: A cohort study. Arthritis Res. Ther..

[50] Wluka A.E., Wang Y., Davis S.R., Cicuttini F.M. (2005). Tibial plateau size is related to grade of joint space narrowing and osteophytes in healthy women and in women with osteoarthritis. Ann. Rheum. Dis..

[51] Bredbenner T.L., Eliason T.D., Potter R.S., Mason R.L., Havill L.M., Nicolella D.P. (2010). Statistical shape modeling describes variation in tibia and femur surface geometry between Control and Incidence groups from the osteoarthritis initiative database. J. Biomech..

[52] Barr A.J., Dube B., Hensor E.M., Kingsbury S.R., Peat G., Bowes M.A., Conaghan P.G. (2014). The relationship between clinical characteristics, radiographic osteoarthritis and 3D bone area: Data from the osteoarthritis initiative. Osteoarthr. Cartil..

[53] Haverkamp D.J., Schiphof D., Bierma-Zeinstra S.M., Weinans H., Waarsing J.H. (2011). Variation in joint shape of osteoarthritic knees. Arthritis Rheum..

[54] Yang Z.Y., Chen W., Li C.X., Wang J., Shao D.C., Hou Z.Y., Gao S.J., Wang F., Li J.D., Hao J.D. (2015). Medial compartment decompression by fibular osteotomy to treat medial compartment knee osteoarthritis: A pilot study. Orthopedics.

[55] Pelletier J.P., Roubille C., Raynauld J.P., Abram F., Dorais M., Delorme P., Martel-Pelletier J. (2015). Disease-modifying effect of strontium ranelate in a subset of patients from the Phase III knee osteoarthritis study SEKOIA using quantitative MRI: Reduction in bone marrow lesions protects against cartilage loss. Ann. Rheum. Dis..

[56] Thambyah A., Goh J.C., De S.D. (2005). Contact stresses in the knee joint in deep flexion. Med. Eng. Phys..

[57] Wirth W., Hellio Le Graverand M.P., Wyman B.T., Maschek S., Hudelmaier M., Hitzl W., Nevitt M., Eckstein F., Group O.A.I.I. (2009). Regional analysis of femorotibial cartilage loss in a subsample from the Osteoarthritis Initiative progression subcohort. Osteoarthr. Cartil..

[58] Cicuttini F., Forbes A., Morris K., Darling S., Bailey M., Stuckey S. (1999). Gender differences in knee cartilage volume as measured by magnetic resonance imaging. Osteoarthr. Cartil..

[59] Ding C., Cicuttini F., Scott F., Cooley H., Jones G. (2005). Knee structural alteration and BMI: A cross-sectional study. Obes. Res..

[60] Borboudakis G., Tsamardinos I. (2021). Extending greedy feature selection algorithms to multiple solutions. Data Min. Knowl. Discov..

