# Supplementary material for: A Machine Learning Model to Predict Knee Osteoarthritis Cartilage Volume Changes over Time Using Baseline Bone Curvature
Source: Biomedicines. 2022 May 26;10(6):1247. doi: 10.3390/biomedicines10061247 (PMC9220338; doi:10.3390/biomedicines10061247)
Supplement: Supplementary file 1 [file biomedicines-10-01247-s001.zip › biomedicines-1690891-supplementary.pdf]

Bonakdari H et al
A machine learning model to predict knee osteoarthritis cartilage volume changes over time using baseline bone curvature

SUPPLEMENTARY MATERIALS

Table S1. The optimal values of the user-defined parameters of the machine learning methods

Method	Parameter	Setting
M5P	The minimum allowable number of instances at a leaf node	21
	The number of decimal places to be used for the output of numbers in the models	24
RF	The number of randomly chosen attributes	10
	Seed	20
	The maximum depth of the tree	5
	The number of decimal places to be used for the output of numbers in the models	4
M5Rules	The minimum number of instances to allow at a leaf node	18
	The number of decimal places to be used for the output of numbers in the models	4
MLP	The learning rates for weight updates	0.3
	Momentum applied to the weight updates	0.2
	Iteration number	10000
	Seed used to initialize the random number generator	100
	The number of decimal places to be used for the output of numbers in the models	4
ANFIS	Iteration number	10000
	Optimal values of initial-increase	1.1
	Step-size-decrease	0.9
	Initial step-size	0.01
	Membership function	Gaussian

M5P, M5 prime; RF, random forest; MLP, multilayer perceptron; ANFIS, adaptive neuro-fuzzy inference system

Table S2. Performance of five machine learning algorithms in predicting cartilage volume loss at one year in 12 regions

	ANFIS			RF			MLP			M5Rules			M5P		
**Outcome**	**R**	**RMSE**	**MAE**	**R**	**RMSE**	**MAE**	**R**	**RMSE**	**MAE**	**R**	**RMSE**	**MAE**	**R**	**RMSE**	**MAE**
**1**	0.78	0.020	0.015	0.72	0.025	0.020	0.65	0.027	0.021	0.70	0.023	0.017	0.64	0.024	0.019
**2**	0.74	0.022	0.015	0.70	0.026	0.020	0.63	0.026	0.019	0.67	0.025	0.018	0.64	0.025	0.019
**3**	0.81	0.021	0.016	0.71	0.028	0.022	0.65	0.028	0.022	0.76	0.025	0.019	0.63	0.028	0.021
**4**	0.86	0.027	0.020	0.74	0.028	0.022	0.59	0.033	0.026	0.67	0.027	0.020	0.61	0.028	0.021
**5**	0.78	0.021	0.015	0.72	0.028	0.022	0.60	0.030	0.024	0.72	0.024	0.019	0.58	0.028	0.021
**6**	0.77	0.023	0.016	0.71	0.029	0.023	0.67	0.028	0.021	0.69	0.027	0.020	0.49	0.031	0.023
**7**	0.78	0.025	0.017	0.67	0.033	0.026	0.68	0.032	0.025	0.72	0.029	0.022	0.56	0.033	0.024
**8**	0.74	0.036	0.026	0.72	0.031	0.024	0.77	0.025	0.019	0.74	0.027	0.020	0.56	0.031	0.024
**9**	0.77	0.027	0.020	0.72	0.034	0.027	0.68	0.035	0.027	0.70	0.031	0.024	0.64	0.032	0.025
**10**	0.78	0.026	0.018	0.70	0.033	0.026	0.68	0.032	0.024	0.71	0.030	0.023	0.64	0.032	0.024
**11**	0.86	0.026	0.018	0.76	0.040	0.030	0.63	0.042	0.032	0.68	0.040	0.029	0.64	0.039	0.029
**12**	0.76	0.057	0.040	0.75	0.038	0.030	0.56	0.051	0.042	0.72	0.036	0.027	0.63	0.038	0.028

Outcome 1, Global knee; 2, Global femur; 3, Global condyles; 4, Global tibial plateau; 5, Lateral compartment; 6, Lateral femur; 7, Lateral condyle; 8, Lateral tibial plateau; 9, Medial compartment; 10, Medial femur; 11, Medial condyle; 12, Medial tibial plateau.
ANFIS, adaptive neuro-fuzzy inference system; RF, random forest; MLP, multilayer perceptron; M5P, M5 prime; R, correlation coefficient; RMSE, root mean square error; MAE, mean absolute error.
